# Prematurity and Long-Term Respiratory Morbidity—What Is the Critical Gestational Age Threshold?

**DOI:** 10.3390/jcm11030751

**Published:** 2022-01-30

**Authors:** Gil Gutvirtz, Tamar Wainstock, Eyal Sheiner, Gali Pariente

**Affiliations:** 1 Department of Obstetrics and Gynecology, Soroka University Medical Center, Ben-Gurion University of the Negev, Beer-Sheva 8457108, Israel; sheiner@bgu.ac.il (E.S.); galipa@bgu.ac.il (G.P.); 2 Department of Public Health, Faculty of Health Sciences, Ben-Gurion University of the Negev, Beer-Sheva 8457108, Israel; wainstoc@bgu.ac.il

**Keywords:** prematurity, gestational age, threshold, respiratory morbidity, pediatric hospitalization

## Abstract

Respiratory morbidity is a hallmark complication of prematurity. Children born preterm are exposed to both short- and long-term respiratory morbidity. This study aimed to investigate whether a critical gestational age threshold exists for significant long-term respiratory morbidity. A 23-year, population-based cohort analysis was performed comparing singleton deliveries at a single tertiary medical center. A comparison of four gestational age groups was performed according to the WHO classification: term (≥37.0 weeks, reference group), moderate to late preterm (32.0–36.6 weeks), very preterm (28.0–31.6 weeks) and extremely preterm (24.0–27.6 weeks). Hospitalizations of the offspring up to the age of 18 years involving respiratory morbidities were evaluated. A Kaplan–Meier survival curve was used to compare cumulative hospitalization incidence between the groups. A Cox proportional hazards model was used to control for confounders and time to event. Overall, 220,563 singleton deliveries were included: 93.6% term deliveries, 6% moderate to late preterm, 0.4% very preterm and 0.1% extremely preterm. Hospitalizations involving respiratory morbidity were significantly higher in children born preterm (12.7% in extremely preterm children, 11.7% in very preterm, 7.0% in late preterm vs. 4.7% in term, *p* < 0.001). The Kaplan–Meier survival curve demonstrated a significantly higher cumulative incidence of respiratory-related hospitalizations in the preterm groups (log-rank, *p* < 0.001). In the Cox regression model, delivery before 32 weeks had twice the risk of long-term respiratory morbidity. Searching for a specific gestational age threshold, the slope for hospitalization rate was attenuated beyond 30 weeks’ gestation. In our population, it seems that 30 weeks’ gestation may be the critical threshold for long-term respiratory morbidity of the offspring, as the risk for long-term respiratory-related hospitalization seems to be attenuated beyond this point until term.

## 1. Introduction

Prematurity is defined by the WHO as a birth before 37 completed weeks’ gestation and is associated with significant infant mortality and morbidity [1,2]. Worldwide, the preterm birth rate is estimated to be approximately 11% [3,4]. The WHO defines three sub-categories for prematurity according to gestational age (GA) at birth: extremely preterm (less than 28 weeks’ gestation), very preterm (28–32 weeks’ gestation) and moderate to late preterm (32–36 weeks’ gestation). This classification mainly reflects the offspring prognosis, as mortality rates correlate with GA [5]. Infants born at or before 28 weeks of gestation have the highest mortality with reported death rates of approximately 50 percent [6], although infant survival rates have improved over the years, especially in high-income countries [7]. Nevertheless, even among tertiary neonatal centers in the United States, there is a variation in mortality that ranges between 5% to 36% for all GAs and 13–73% for infants less than 25 weeks’ gestation [8]. One of the main causes of early infant death is respiratory disorders [9,10] especially in extremely preterm infants. Preterm birth interferes with the development of the lung and may render it less effective as a gas exchanger or may make it more susceptible to disease by changing the “program” that determines its development [11]. Those who survive may suffer short-term complications (e.g., respiratory distress syndrome (RDS), patent ductus arteriosus (PDA) and bronchopulmonary dysplasia (BPD)) during the neonatal period [12] and long-term sequelae including repeated hospitalizations [13,14], chronic respiratory diseases [15,16,17] and neurodevelopmental disabilities such as cerebral palsy (CP) [18,19].

Respiratory morbidity is one of the most prominent complications of prematurity, starting with RDS with increasing incidence as GA decreases [12] and followed by BPD as a later respiratory complication. While these complications are short-term in appearance, they are known to have long-term consequences on lung function [20], and the outcome of poor lung development depends on the type and severity of the insult as well as the lung developmental stage at which it occurs [21].

As respiratory morbidity is one of the devastating outcomes of prematurity, we opted to investigate the correlation between the degree of prematurity and long-term respiratory morbidity of the offspring in order to establish a critical cut-off at which the long-term respiratory morbidity of the offspring would be higher.

## 2. Methods

This was a population-based, retrospective study conducted at the Soroka University Medical Center (SUMC) between the years 1991 and 2014. It included all singleton deliveries occurring within this time period at SUMC and followed offspring hospitalizations in SUMC until 18 years of age.

The institutional review board (SUMC IRB Committee) approved the study that has been performed, which is in accordance with the ethical standards laid down by the 1964 Declaration of Helsinki and its later amendments (Helsinki Declaration 1975, revision 2013).

The SUMC is the largest birth center in the country and the sole tertiary hospital in the Negev area that includes a neonatal intensive care unit (NICU) and pediatric wards.

The area has experienced positive immigration over the last two decades, with increasing annual birth rates reaching approximately 15,000 by the end of the study period. As the only hospital in the area, children born in the SUMC are also expected to be hospitalized in the hospital’s pediatric wards, if indicated.

A comparison of 4 gestational age groups was performed according to the WHO classification [1]: term births (≥37.0 weeks), moderate to late preterm (32.0–36.6 weeks), very preterm (28.0–31.6 weeks) and extremely preterm (24.0–27.6 weeks).

We excluded cases of multiple pregnancies and congenital malformations, as both factors are associated with preterm delivery and chronic health problems. Perinatal mortality cases were also excluded from the long-term analysis.

Maternal characteristics included maternal age and background morbidity (pre-gestational diabetes and chronic hypertension). Selective pregnancy complications were also recorded including gestational diabetes mellitus (GDM) and hypertensive disorders (preeclampsia with and without severe features and eclampsia).

The primary outcome variables included respiratory-related hospitalizations of the offspring up to the age of 18 years, as recorded in hospital records, using predefined diagnoses of ICD-9 codes detailed in the Table S1. Secondary outcomes assessed included adverse perinatal outcomes such as intra-amniotic infection rates (a clinical diagnosis made by an obstetrician during labor defined by a combination of common clinical criteria for chorioamnionitis, including maternal fever, maternal and fetal tachycardia, uterine tenderness and foul amniotic fluid), low Apgar scores (<7) given to the neonate at 5 min, small for gestational age (SGA), defined as birthweight <5th percentile for gestational age and gender, and cesarean delivery rates.

Follow-up time was defined as time to event (respiratory-related hospitalization, as first diagnosis or background diagnosis) and ended at either first hospitalization, child death (during hospitalization for unrelated morbidity), reaching age of 18 or at the end of the study period, whichever milestone was reached first.

We used 2 distinct hospital databases in order to merge and cross-link maternal and offspring data The first is the computerized obstetrics and gynecology department database, which consists of maternal and obstetrical information recorded at the mothers’ admission to the delivery room after assessing medical prenatal care records and immediately following delivery by an obstetrician. The second is the pediatric hospitalization database, which includes demographic information and ICD-9 codes for all medical diagnoses made during hospitalization in any of the pediatric departments at the SUMC. In the SUMC, all records are routinely reviewed by experienced medical secretaries for accuracy and completeness prior to entering them into the databases.

### Statistical Analysis

We used the SPSS package 23rd edition (IBM/SPSS, Chicago, IL, USA) to perform the study’s statistical analysis. Background, clinical and outcome variables were compared between the study groups using χ^2^ tests for categorical variables and Student’s *t* test for continuous variables with normal distribution. Kaplan–Meier survival curves were used to compare cumulative respiratory-related hospitalization incidences during follow-up time according to gestational age at birth, divided into the four subgroups characterized above, and differences between curves were assessed using log-rank test. A Cox regression model was used to investigate the association between gestational age at birth and pediatric respiratory-related hospitalization risk. The model was adjusted based on the univariate analysis and clinically relevant variables including follow-up time, maternal age, mode of delivery, birthweight, diabetes mellitus, hypertensive disorders and presence of intra-amniotic infection (clinically diagnosed chorioamnionitis). Term deliveries were used as the reference group for the comparison. All analyses were two-sided. A *p*-value < 0.05 was considered statistically significant.

## 3. Results

During the study period, 220,563 singleton deliveries met the inclusion criteria. Of those, 93.6% (*n* = 206,361) were born at term, 6% (*n* = 13,308) were moderate to late preterm, 0.4% (*n* = 776) were very preterm and 0.1% (*n* = 118) were extremely preterm.

Maternal characteristics and immediate perinatal outcomes according to the different gestational age groups are presented in Table 1. A statistically significant difference in maternal age was noted between groups; however, it was clinically irrelevant. Incidence of hypertensive disorders was highest in women who delivered very preterm and lowest in women who delivered at term. Incidence of intra-amniotic infection and cesarean deliveries decreased progressively as gestational age increased. Diabetes was more common in women from the preterm groups except for those who delivered extremely preterm.

Table 2 summarizes the selected respiratory morbidities and total hospitalization rate for the four study groups according to prematurity severity. Offspring born preterm had a significantly higher respiratory-related hospitalization rate compared to term offspring, as the hospitalization rate increased with decreasing gestational age (4.7%, 7.0%, 11.7% and 12.7% for offspring born in term delivery, moderate to late PTB, very PTB and extremely PTB, respectively, *p* < 0.001 for trends).

Mean age at hospitalization for offspring born at 24–28 weeks’ gestational age was 10.2 +/− 6.6 years. A total of 12.7% of the cases occurred by the age of 1 year, and 30.5% by the age of 5 years. The median age was 12.1 years. For offspring born at 28–32 weeks’ gestational age, the mean age at hospitalization was 9.13 +/− 6.4 years. A total of 11.9% of the cases occurred by the age of 1 year, and 37% by the age of 5. Median age was 8.65 years. For offspring born at 32–36 weeks’ gestational age, the mean age at hospitalization was 9.92 +/− 6.1 years. A total of 7.5% of the cases occurred by the age of 1 year, and 28.1% by the age of 5. The median age was 10.2 years.

The mean age at hospitalization for offspring born at term was 10.02 +/− 6.1 years. A total of 6.8% of the cases occurred by the age of 1 year, and 27.5% by the age of 5. The median age was 10.2 years.

A significant difference was noted in the age of hospitalization between age groups 28–32 and 32–36 (*p* < 0.001) and between 28–32 and term (*p* < 0.001).

**Figure 1:**
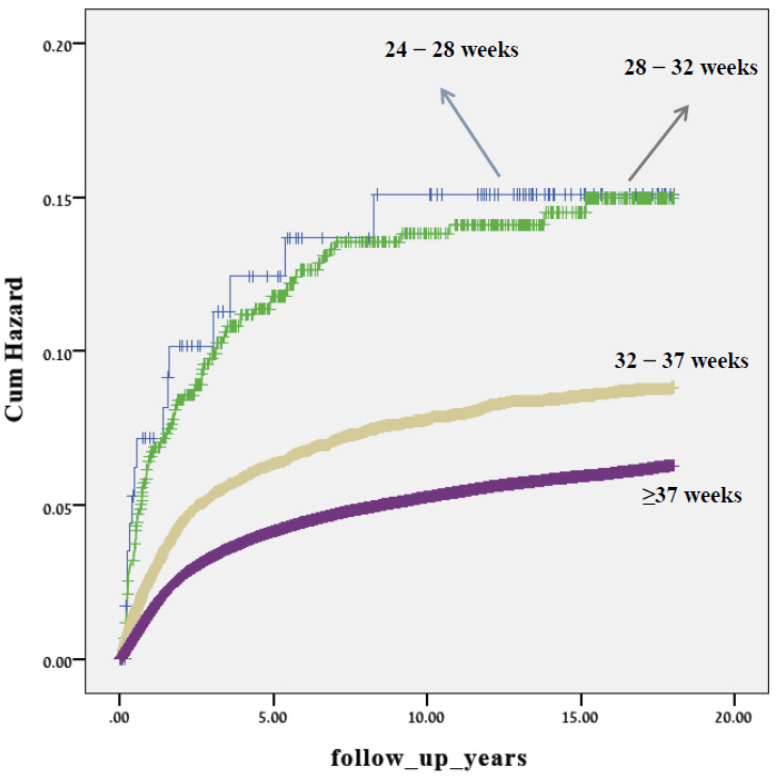
A Kaplan–Meier survival curve demonstrating the cumulative incidence of respiratory-related hospitalizations among study groups of different gestational age at birth.

Presented in Figure 1 are the Kaplan–Meier survival curves showing a progressively higher cumulative incidence of offspring respiratory-related hospitalizations as gestational age decreases. Similar survival curves of offspring born extremely and very preterm were noted, with the highest cumulative hospitalization incidence. For all gestational age groups, the sharpest incline of hospitalizations was seen by the age of 5.

**Figure 2:**
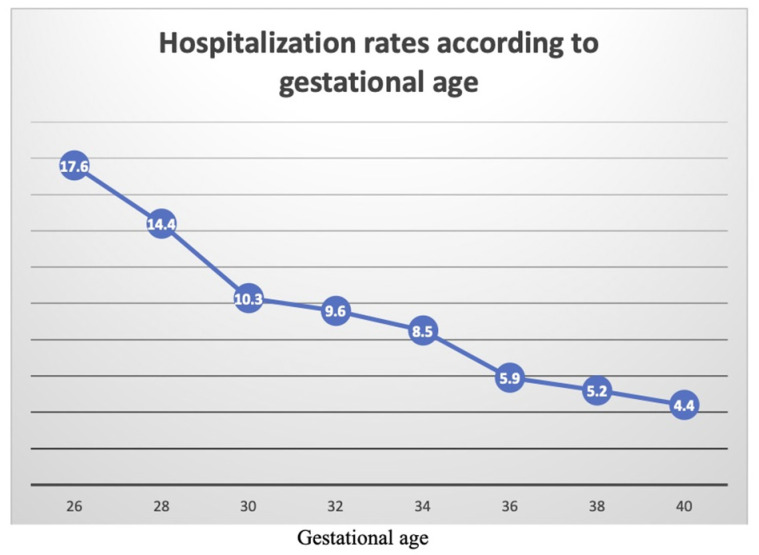
Respiratory-related hospitalization rates according to gestational age.

A univariate analysis of respiratory-related hospitalization rates according to week of gestation at birth is presented in Figure 2. A general inverse relationship can be seen between gestational week at birth and incidence of respiratory-related hospitalization. At 30 weeks’ gestation, the hospitalization rate drops significantly to approximately 10% (as opposed to 17% at 26 weeks); then, it is attenuated until 34 weeks, with a further decrease in hospitalization rate at 36 weeks. The curve continues to drop to its lowest point at 40 weeks (full term), although the decline is less prominent.

A Cox proportional hazards model is presented in Table 3. The model was constructed to adjust for follow-up time, and control for statistically significant and clinically relevant variables is described in the methods section. Using this model, prematurity (birth before 37 completed weeks) was found to be independently associated with long-term respiratory morbidity. Compared to term delivery, birth before 32 weeks’ gestation (whether extremely or very preterm) carries a 2.0-fold risk for long-term respiratory morbidity, while birth at moderate to late preterm (32.0–36.6 weeks) carries a 1.3-fold risk for long-term respiratory morbidity. The critical threshold beyond which long-term respiratory morbidity decreased is 30 days.

Repeated hospitalizations of the offspring were also evaluated. We found the mean re-hospitalization rate decreased with increasing gestational age, from 20% in extremely premature infants (24–28 weeks’ gestation) to 16% in the very preterm group (28–32 weeks), 10% in the moderate to late preterm group (32–37 weeks) and finally decreasing to only 6% in term infants (≥37 weeks).

**Figure 3:**
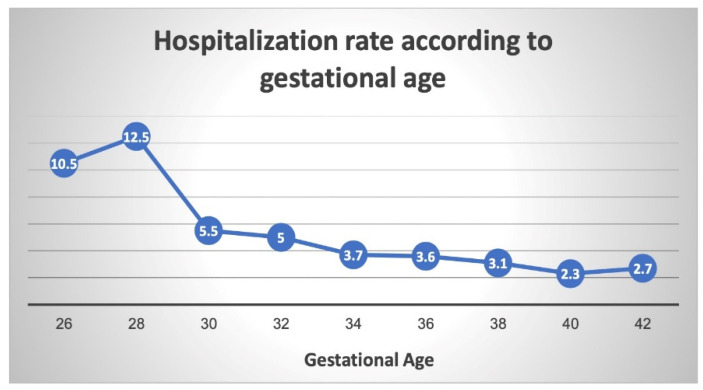
Respiratory-related hospitalization rates according to gestational age (offspring born before the year 2000).

Finally, we conducted a sub-analysis of the original cohort, dividing it to two distinct time periods—offspring born before the year 2000 (1991–1999) and those born after (2000–2014). In offspring born before the year 2000, with the exception from 26 to 28 weeks, an inverse relationship can be seen between gestational week at birth and incidence of respiratory-related hospitalization (Figure 3). At 30 weeks’ gestation, the hospitalization rate is more than halved to 5.5% from 12.5% at 28 weeks. From 30 weeks’ gestation, the hospitalization rate slowly declines to its lowest rate at full term (40 weeks).

A Cox regression model for this cohort, adjusted for maternal age, mode of delivery, birthweight, diabetes mellitus, hypertensive disorders and presence of intra-amniotic infection (clinically diagnosed chorioamnionitis), found prematurity (birth before 37 completed weeks) was independently associated with long-term respiratory morbidity. Compared to term delivery, being born extremely preterm, very preterm and moderate to late preterm carries a significant higher risk for long-term respiratory morbidity (aHR of 3.33, 2.61 and 1.32, respectively, Table 4).

**Figure 4:**
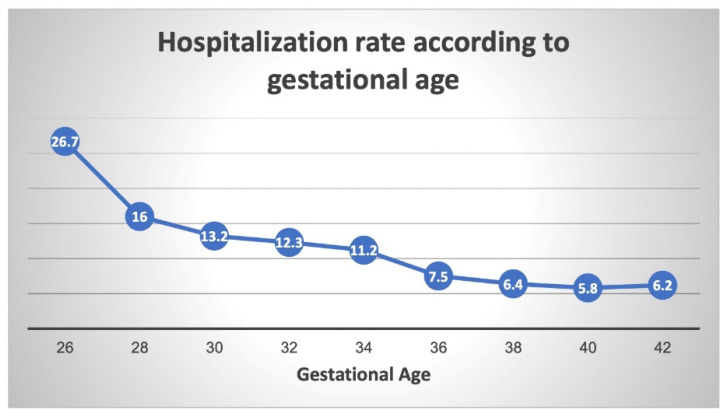
Respiratory-related hospitalization rates according to gestational age (offspring born after the year 2000).

In offspring born after the year 2000, a general inverse relationship can once again be seen between gestational week at birth and incidence of respiratory-related hospitalization (Figure 4). The gradual decline in the hospitalization rate is attenuated from 30 weeks’ gestation until 34 weeks, with a further decrease in the hospitalization rate at 36 weeks. The curve continues to drop to its lowest point at full term (40 weeks).

A similar Cox regression model for this sub-analysis, adjusted for maternal age, mode of delivery, birthweight, diabetes mellitus, hypertensive disorders and presence of intra-amniotic infection (clinically diagnosed chorioamnionitis), also found prematurity (birth before 37 completed weeks) was independently associated with long-term respiratory morbidity. Compared to term delivery, being born extremely preterm, very preterm and moderate to late preterm carries a significant higher risk for long-term respiratory morbidity (aHR of 2.04, 1.97 and 1.32, respectively, Table 5).

## 4. Discussion

This large retrospective cohort study examined the long-term incidence of respiratory-related hospitalizations of children born preterm, stratified by the severity of prematurity and compared to offspring born at term. We found the risk for long-term respiratory morbidity to be increased as gestational age at birth decreased, which is in accordance with earlier literature [22,23,24]. Nevertheless, while most studies associate more extremely prematurity with higher rates of respiratory morbidity, they could not point out the gestational age threshold that carries the highest risk, after which the prognosis improves. Using a sub-analysis of hospitalization rates according to gestational age at birth, we found the sharpest decline at 30 weeks, which then attenuates until reaching near-term (36 weeks), where it once again declines to reach its lowest point at full term (40 weeks). 30 weeks’ gestation seems to be the threshold where the risk for long-term respiratory morbidity is significantly reduced and from which point the hospitalization rate decreases to less than 10%.

This threshold is similar to that found by Copper et al. for preterm infants’ survival rates. Their study, conducted in the U.S., reported that by 30 weeks of gestation, survival rates were >90% and increased by <1% per week thereafter until term [25], which is in line with our finding that this threshold is an important developmental stage for the preterm newborn.

The human fetal lung, like other crucial organs, has a developmental timeline that progresses from the embryonic period until birth. The pulmonary alveoli, the definitive units of gas exchange, and its supportive structures develop during late fetal and early postnatal life. Postnatally, the lungs continue to mature as alveolarization and microvascular maturation continue until a “complete” functional lung is achieved [26]. Abrupt discontinuation of lung development process (i.e., preterm birth) results in a premature and immature functional lung, commonly presenting as neonatal respiratory distress syndrome (RDS) and its later complication, bronchopulmonary dysplasia (BPD), with increased incidence and severity with decreasing gestational age [12,27]. The primary cause of RDS is deficiency of pulmonary surfactant, which is developmentally regulated and starts at around 20 weeks’ gestation [28]. However, most alveolar surfactant is produced after about 30–32 weeks’ gestation, and preterm infants born before then will probably develop RDS [29], as both a decrease in the quantity and quality of surfactant contributes to decreased surfactant activity, resulting in RDS. Importantly, the characteristic clinical course of RDS is observed less frequently because of interventions that reduce the risk of RDS, including the use of antenatal glucocorticoid therapy, early intubation for surfactant therapy, and/or respiratory support for the preterm neonate [30]. Nevertheless, RDS is very common in extremely and very preterm infants, and even those born between 30 and 34 weeks have a significant risk for acute respiratory disease, as reported in a Swedish study in which approximately 30% of the moderate to late preterm infants had this complication [31]. As a result, it is common for preterm infants, especially those born very preterm, to be exposed to high levels of oxygen for extended periods owing to respiratory insufficiency. It is now known from multiple animal studies that prolonged inhalation of high levels of oxygen can permanently alter lung development, especially the formation of alveoli [21,32,33]. Preterm infants are more susceptible to oxygen toxicity compared with term infants due to their more immature antioxidant enzyme systems [28,34]. Other than oxygen toxicity, preterm infants may also suffer injury caused by mechanical ventilation (volutrauma) [35,36,37]. For extremely preterm infants who receive mechanical ventilation, the risk of BPD is high [38]. Because of the strong evidence that aggressive mechanical ventilation plays a major role in the pathogenesis of BPD, management of preterm infants requiring respiratory support has moved towards initial noninvasive measures. However, despite the use of noninvasive respiratory support, up to 50 percent of extremely preterm infants are eventually intubated and mechanically ventilated [39]. Extended ventilatory support carries an increased risk not only for BPD but also for later airway problems such as asthma and chronic obstructive pulmonary disease (COPD) [40]. Looking at chronic respiratory morbidities, retrospective studies and meta-analyses found prematurity to be an important risk factor for childhood wheezing disorders, specifically asthma [41,42,43]. In a large Swedish study, children born extremely preterm had a four-times higher risk of asthma compared to those born at term [44]. Furthermore, studies of children and young adults born moderately to very preterm show persistent and significant lung function deficits [17] and reduced exercise capacity in those born very preterm [45], all of which imply the significant health consequences of prematurity. In light of the evidence regarding the lung prematurity at extremely and very preterm gestational ages, our results can be easily interpreted. Interestingly, our study provides similar results to an earlier study carried out by our group [46], which also found that children born before 32 weeks’ gestation were at a significantly higher risk for infectious morbidity, including respiratory infections, once again reinforcing the thesis that a premature lung is more susceptible to respiratory morbidity later in life.

Our study found significant higher rates of intrauterine infection in children born preterm, specifically in the extremely preterm group. This factor is known to affect infants’ lung function and health [47]. Hence, we included it in the Cox regression models and found that it did not change the results of the study. As expected, rates of induced labor were far less common in the extremely and very preterm groups but still reached around 10% of deliveries in these groups. This might have resulted from the much higher rate of intrauterine infections that forced the attending obstetricians to induce labor in these early gestational ages. The same may be hypothesized due to the increased rates of hypertensive disorders found in the extremely and very preterm groups, though to a much lesser extent. Compared to term deliveries, diabetes was also more common in women who delivered very and moderate to late preterm, although this was not the case for those who delivered extremely preterm (probably since the gestational age of extreme prematurity is set before the recommended gestational age for GDM screening). Nevertheless, we also included these variables in the Cox model to find the results were unchanged. Finally, as expected, cesarean delivery rates were also significantly higher in the extremely and very preterm group. As this mode of delivery is known to affect long-term respiratory morbidity of the offspring even at term [48], we also included this variable as a possible confounder in the multivariant model. Even after controlling for all these possible confounders, the Cox model shows that being born preterm carries a significant risk for long-term respiratory morbidity, which correlates to the severity of prematurity.

The strength of this study stems from its large cohort and the long-term follow-up on children born in a single tertiary medical center. Being the sole hospital in the Negev region, we were able to combine databases from the Obstetrics and Pediatric wards to match the data for the offspring, assuming that if the child required hospitalization, it would have occurred in our institute. Since the Negev region has enjoyed positive immigration rates for the last 2 decades, this assumption is plausible. Maternal and obstetrical information was recorded by obstetricians assessing the mothers’ medical prenatal care records immediately following delivery. Prior to archiving, all records were also reviewed by trained medical secretaries for accuracy and completeness of the information. Naturally, human errors are inevitable, but considering the large cohort of patients, we assume these are very marginal and statistically insignificant.

This was a retrospective study and as such, it has inherent flaws that allowed us only to assume an association between exposure (prematurity) and outcome (long-term morbidity) and not a causative explanation. Furthermore, many other factors could have led to the increased risk of long-term respiratory morbidity, rather than prematurity alone. We acknowledge that our dataset lacks some important factors for children long-term respiratory prognosis such as the treatments provided during pregnancy, offspring hospitalization or other post-discharge medical care. An important limitation to fully interpreting our results is the lack of data regarding the use of antenatal corticosteroid therapy. Dozens of randomized trials have confirmed that a course of antenatal corticosteroid therapy administered to women at risk for preterm delivery reduced the incidence and severity of respiratory distress syndrome (RDS) and mortality in offspring [49]. However, since the use of antenatal corticosteroid therapy for preterm delivery was already well established during the study period, we assume most patients in our cohort who presented with preterm labor or were induced to deliver in early gestational ages received the treatment non-selectively. Another important limitation of this study is the difficulty to account for some postnatal variables such as socioeconomic status and environmental influences, which might affect offspring respiratory health [50]. Our long-term database is also based on hospitalization records, and as such it includes only cases that were severe enough to require in-hospital treatment. Milder cases of respiratory morbidity that were treated in ambulatory setting were out of our reach. However, we assume that our results are actually an underestimation of the true prevalence of long-term respiratory morbidity in extremely and very preterm infants. Finally, this study evaluated long-term morbidity of offspring until 18 years of age and did not include longer-term follow-ups into adulthood, as a longer-term follow-up was not in the scope of this study. More studies investigating the longer-term follow-up of respiratory-related morbidity of the offspring according to gestational age would help to shed some more light on the true prevalence of gestational-age-related respiratory diseases of the total population.

## 5. Conclusions

Although the respiratory morbidity of a preterm infant is well described in the literature, most studies reported the inverse relationship between morbidity and gestational age but could not point out the exact gestational age threshold after which long-term morbidity is reduced. Our study shows 30 weeks’ gestation to be an important milestone that can serve obstetricians and neonatologist in further calculating the risk and counselling patients on the long-term respiratory morbidity of a preterm infant. Obstetricians may also use our data when considering the consequences of preterm induction of labor for patients presenting with pregnancy complications, as we have demonstrated the risk of respiratory morbidity by gestational age. Our data also provide some reassurance for pediatricians and patients in the study cohort of hospitalized patients with respiratory morbidity, as most morbidities investigated here were found at relatively low rates.

## Figures and Tables

**Table 1:** Maternal characteristics and pregnancy outcomes according to gestational age.

Maternal Characteristics	Extremely Preterm (24.0–27.6) N = 118	Very Preterm (28.0–31.6) N = 776	Moderate to Late Preterm (32.0–36.6) N = 13,308	Term Delivery (≥37.0) N = 206,361	*p* Value ^a^
Maternal age (mean ± SD, years)	28.4 ± 6.3	28.3 ± 6.3	28.3 ± 6.2	28.2 ± 5.7	<0.001
Diabetes mellitus ^b^ (%)	0.0	6.2	8.2	5.3	<0.001
Hypertensive disorders ^c^ (%)	8.5	19.1	12.9	4.7	<0.001
Induction of labor (%)	10.2	9.3	23.1	27.6	<0.001
Intra-amniotic infection (%)	33.1	14.3	2.6	0.3	<0.001
Cesarean Delivery (%)	51.7	52.8	31.0	12.7	<0.001
Low Apgar at 5 min (<7) (%)	14.4	6.2	2.5	1.4	<0.001
Gestational age at delivery (mean ± SD, weeks)	26.4 ± 0.7	30.0 ± 1.0	35.2 ± 1.1	39.4 ± 1.2	<0.001
Birthweight (mean ± SD, grams)	1096 ± 601	1644 ± 633	2540 ± 495	3270 ± 445	<0.001
SGA ^d^ (%)	3.4	1.8	3.6	4.4	<0.001

^a^ Calculated for all groups using the chi square test for trends; ^b^ including pre-gestational and gestational diabetes; ^c^ including chronic hypertension, gestational hypertension, preeclampsia with or without severe features and eclampsia; ^d^ SGA = small for gestational age, defined as birthweight <5th percentile for gestational age and gender.

**Table 2:** Selected long-term respiratory morbidities in children (up to the age of 18 years) according to gestational age at birth.

Respiratory Morbidity	Extremely Preterm (24.0–27.6) N = 118	Very Preterm (28.0–31.6) N = 776	Moderate to Late Preterm (32.0–36.6) N = 13,308	Term Delivery (≥37.0) N = 206,361	*p* Value ^a^
Asthma (%)	3.4	5.2	3.4	2.5	<0.001
Pleural disease (%)	0.0	0.4	0.1	0.1	0.022
Obstructive sleep apnea (OSA) (%)	1.7	1.3	0.9	0.7	0.002
Other * (%)	8.5	6.6	3.0	1.9	<0.001
Total respiratory hospitalizations (%)	12.7	11.7	7.0	4.7	<0.001

^a^ Calculated for all groups using the chi square test for trends. * Detailed in the supplementary table.

**Table 3:** Multivariable analysis of long-term risk for respiratory-related hospitalizations according to gestational age.

Gestational Age	Adjusted Hazard Ratio (aHR) *	Confidence Interval (95%)	*p* Value
Term delivery (reference) (37–42 weeks)	1	-	-
Moderate to late preterm (32–37 weeks)	1.29	1.20–1.39	<0.01
Very preterm (28–32 weeks)	2.02	1.62–2.52	<0.01
Extremely preterm (24–28 weeks)	2.04	1.21–3.44	<0.01

* Adjusted for maternal age, birthweight, diabetes mellitus, hypertensive disorders, presence of intra-amniotic infection and mode of delivery.

**Table 4:** Multivariable analysis of long-term risk for respiratory-related hospitalizations according to gestational age (offspring born before the year 2000).

Gestational Age	Adjusted Hazard Ratio (aHR) *	Confidence Interval (95%)	*p* Value
Term delivery (reference) (37–42 weeks)	1	-	-
Moderate to late preterm (32–37 weeks)	1.32	1.12–1.56	<0.01
Very preterm (28–32 weeks)	2.61	1.70–4.02	<0.01
Extremely preterm (24–28 weeks)	3.33	1.50–7.39	<0.01

* Adjusted for maternal age, birthweight, diabetes mellitus, hypertensive disorders, presence of intra-amniotic infection and mode of delivery.

**Table 5:** Multivariable analysis of long-term risk for respiratory-related hospitalizations according to gestational age (offspring born after the year 2000).

Gestational Age	Adjusted Hazard Ratio (aHR) *	Confidence Interval (95%)	*p* Value
Term delivery (reference) (37–42 weeks)	1	-	-
Moderate to late preterm (32–37 weeks)	1.32	1.21–1.44	<0.01
Very preterm (28–32 weeks)	1.97	1.52–2.55	<0.01
Extremely preterm (24–28 weeks)	2.04	1.01–4.12	0.04

* Adjusted for maternal age, birthweight, diabetes mellitus, hypertensive disorders, presence of intra-amniotic infection and mode of delivery.

## Supplementary Materials

The following supporting information can be downloaded at: https://www.mdpi.com/article/10.3390/jcm11030751/s1. Table S1: Respiratory morbidity ICD-9 codes.
