# Supplementary material for: Prematurity and Long-Term Respiratory Morbidity—What Is the Critical Gestational Age Threshold?
Source: J Clin Med. 2022 Jan 30;11(3):751. doi: 10.3390/jcm11030751 (PMC8836586; doi:10.3390/jcm11030751)
Supplement: Supplementary file 1 [file jcm-11-00751-s001.zip › jcm-1551778-supplementary.pdf]

Table S1. ICD-9 Codes for pediatric respiratory morbidity

GROUPS	DIAG. CODE	DIAGNOSIS DESCRIPTION
**ASTHMA**	49300	EXTRINSIC ASTHMA, UNSPECIFIED
	49320	CHR. OBSTRUCTIVE ASTHMA, UNSPECIFIED
	49321	CHR. OSBTRUCTIVE ASTHMA WITH STATUS ASTHMATICUS
	49390	ASTHMA, UNSPECIFIED
	49390	ASTHMA, UNSPECIFIED TYPE, WITHOUT MENTION OF STATUS ASTHMATICUS
	49390	ASTHMA, UNSPECIFIED TYPE, WITHOUT MENTION OF STATUS ASTHMATICUS OR ACUTE EXACERBATION OR UNSPECIFIED
	49391	ASTHMA, UNSPECIFIED TYPE, WITH STATUS ASTHMATICUS
	49392	UNSPECIFIED ASTHMA WITH (ACUTE) EXACERBATION
	496	CHRONIC AIRWAY OBSTRUCTION, NOT ELSEWHERE CLASSIFIED
**STRUCTURAL - EMPHYSEMA**	4920	EMPHYSEMATOUS BLEB
	4928	OTHER EMPHYSEMA
	5100	EMPYEMA WITH FISTULA
	5109	EMPYEMA WITHOUT MENTION OF FISTULA
	5181	INTERSTITIAL EMPHYSEMA
**BRONCHIECTASIS - FIBROSIS - HEMOSIDEROSIS**	494	BRONCHIECTASIS
	4940	BRONCHIECTESIS WITHOUT ACUTE EXACERBATION
	4941	BRONCHIECTASIS WITH ACUTE EXACERBATION
	4959	UNSPECIFIED ALLERGIC ALVEOLITIS AND PNEUMONITIS
	515	POSTINFLAMMATORY PULMONARY FIBROSIS
	5160	PULMONARY ALVEOLAR PROTEINOSIS
	5161	IDIOPATHIC PULMONARY HEMOSIDEROSIS
**PNEUMONITIS**	5070	PNEUMONITIS DUE TO INHALATION (FOOD,VOMITUS,OR N.O.S.)
	5070	PNEUMONITIS DUE TO INHALATION OF FOOD OR VOMITUS
	5071	PNEUMONITIS DUE TO INHALATION OF OILS AND ESSENCES
	5078	PNEUMONITIS DUE TO OTHER SOLIDS AND LIQUIDS
	5130	ABSCESS OF LUNG
	5168	OTHER SPECIFIED ALVEOLAR AND PARIETOALVEOLAR PNEUMONOPATHIES
	5183	PULMONARY EOSINOPHILIA
	5192	MEDIASTINITIS
	5193	OTHER DISEASES OF MEDIASTINUM, NOT ELSEWHERE CLASSIFIED
**PLEURAL**	5110	PLEURISY WITHOUT MENTION OF EFFUSION OR CURRENT TUBERCULOSIS
	5118	OTHER SPECIFIED FORMS OF PLEURAL EFFUSION, EXCEPT TUBERCULOUS
	5119	UNSPECIFIED PLEURAL EFFUSION
	5120	SPONTANEOUS TENSION PNEUMOTHORAX
	5128	OTHER SPONTANEOUS PNEUMOTHORAX
	51181	MALIGNANT PLEURAL EFFUSION
	51189	OTHER SPECIFIED FORMS OF EFFUSION, EXCEPT TUBERCULOUS
**OBSTRUCTIVE SLEEP APNEA (OSA)**	32723	OBSTRUCTIVE SLEEP APNEA (ADULT)(PEDIATRIC)
	32727	CENTRAL SLEEP APNEA IN CONDITIONS CLASSIFIED ELSEWHERE
	78051	INSOMNIA WITH SLEEP APNEA
	78051	INSOMNIA WITH SLEEP APNEA, UNSPECIFIED
	78057	OTHER AND UNSPECIFIED SLEEP APNEA

	78057	UNSPECIFIED SLEEP APNEA
**OTHER**	514	PULMONARY CONGESTION AND HYPOSTASIS
	5178	LUNG INVOLVEMENT IN OTHER DISEASES CLASSIFIED ELSEWHERE
	518	OTHER DISEASES OF LUNG
	5180	PULMONARY COLLAPSE
	5185	PULMONARY INSUFFICIENCY FOLLOWING TRAUMA AND SURGERY
	5186	ALLERGIC BRONCHOPULMONARY ASPERGILLOSIS
	51883	CHRONIC RESPIRATORY FAILURE
	51900	TRACHEOSTOMY COMPLICATION, UNSP.
	51909	OTHER TRACHEOSTOMY COMPLICATIONS
	5191	OTHER DISEASES OF TRACHEA AND BRONCHUS, NOT ELSEWHERE CLASSIF.
	5198	OTHER DISEASES OF RESPIRATORY SYSTEM, NOT ELSEWHERE CLASSIFIED
	5199	UNSPECIFIED DISEASE OF RESPIRATORY SYSTEM
	51911	ACUTE BRONCHOSPASM
	51919	OTHER DISEASES OF TRACHEA BRONCHUS
	786	SYMPTOMS INVOLVING RESPIRATORY SYSTEM & OTHER CHEST SYMPTOMS
	78607	WHEEZING
	78609	OTHER DYSPNEA AND RESPIRATORY ABNORMALITY
	7990	ASPHYXIA
	7990	ASPHYXIA AND HYPOXEMIA
	9973	RESPIRATORY COMPLICATIONS, NOT ELSEWHERE CLASSIFIED
	99739	OTHER RESPIRATORY COMPLICATIONS
